# Supplementary material for: The Wave Model of Sleep Dynamics and an Invariant Relationship between NonREM and REM Sleep
Source: Clocks Sleep. 2023 Nov 17;5(4):686–716. doi: 10.3390/clockssleep5040046 (PMC10660848; doi:10.3390/clockssleep5040046)
Supplement: Supplementary file 1 [file clockssleep-05-00046-s001.zip › clockssleep-2646600-supplementary.pdf]

**Kharchenko V. and Zhdanova I.V. The wave model of sleep dynamics and an invariant relationship between NonREM and REM sleep**

**Supplemental materials:**

**Supplemental Table 1 The Wave Model of Sleep parameters for groups with Regular and Extended sleep.**

| Group | $U_s$ width $\boldsymbol{\sigma}$ | Number of initial level $\boldsymbol{j_{in}}$ | Resonance level $\boldsymbol{j_c}$ | Resonance width $\boldsymbol{\Gamma}$ |
|---|---|---|---|---|
| Regular sleep | 10 | 10.3 | 7.1 | 0.44 |
| Extended sleep | 11.3 | 11.8 | 7.6 | 0.52 |

Regular sleep: 9-h regular nighttime sleep opportunity, described in Method 16.
Extended sleep: 14-h extended nighttime sleep opportunity, described by Barbato and Wher[30].
At sleep onset, $\varepsilon$ can slightly deviate from exact $U_s$ level, hence $j_{in}$ in model approximation appears non-integer. However, at $x_o$ (Fig. 2) the wavepacket would occupy the closest integer level. Time-scaling constants to convert relative duration values to absolute time (min) and for these groups, $A_{NREM}$ and $A_{REM}$ were, respectively, 0.6 and 23 for Regular sleep and 0.5 and 22 for Extended sleep.

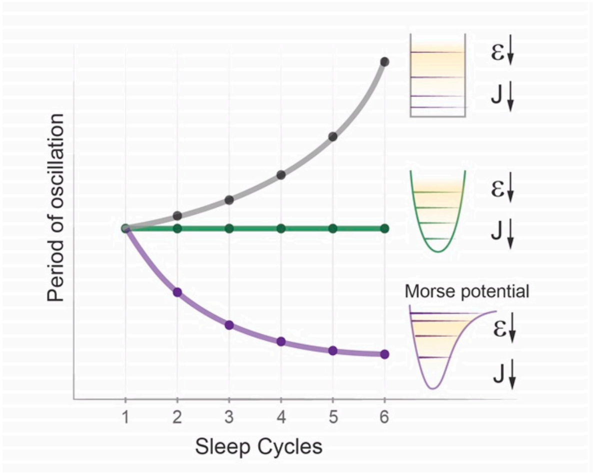


**Figure S1 Consecutive periods of energy relaxation in potential wells of different shape.**
The dynamics of energy $\varepsilon$ relaxation over consecutive cycles toward the lowest energy level j depends on the shape of the potential well and energy gaps between levels. The period of oscillation is inverse proportional to the energy gap. For rectangular potential (black), top-to-bottom reduction of energy gaps leads to gradual increase in cycle period during energy decline. For a parabolic well (green), equal energy gaps lead to periods of equal duration (harmonic oscillator). For asymmetric Morse potential (purple), gradual top-to-bottom increase in energy gaps leads to a decline in the period of oscillations during energy relaxation.

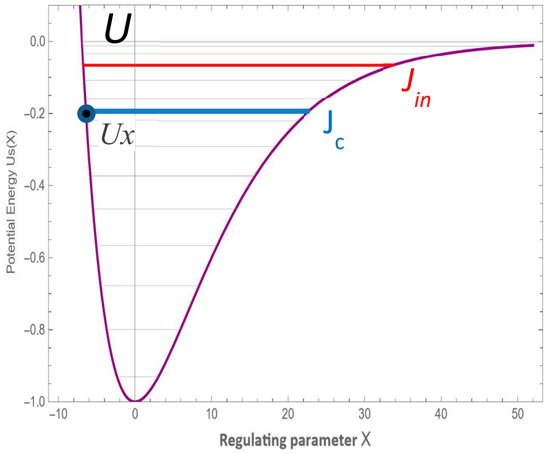


**Figure S2 Actual position of the initial and resonance levels within the *Us* potential well.**
For regular sleep (Fig. 3b, Table 1. Regular), typical position of the initial *Us* energy level ($J_{in}$, red line) of sleep onset is around level 10. The energy of the resonance level (*Jc* blue line) is around *Us* levels 7, near the homeostatic energy threshold, $Ux_c$ (black dot). Note that *Jc* does not belong to *Us* and can be positioned asymmetrically relative to *Us* levels (thin horizontal lines). Conventionally, energy levels are counted from the bottom of the potential well. There is a positive correlation between the total number of *Us* levels and the width of the potential well. In cases of regular sleep, the potential well typically includes around 14 levels.
